# Accuracy of Breast MRI for Surgical Planning After Neoadjuvant Therapy for Patients with Invasive Lobular Carcinoma

**DOI:** 10.1245/s10434-025-17735-6

**Published:** 2025-06-27

**Authors:** Anna Vertido, Tesia McKenzie, Alisha Othieno, Astrid Quirarte, Mandeep Kaur, Mary Kathryn Abel, Maggie Chung, Amie Y. Lee, Rita A. Mukhtar

**Affiliations:** 1 Division of Surgical Oncology, Department of Surgery, University of California San Francisco, San Francisco, CA USA; 2 Division of Breast Surgery, Riverview Medical Center, Hackensack Meridian Health, Shrewsbury, NJ USA; 3 Department of Obstetrics and Gynecology, Kaiser Permanente Roseville Medical Center, Roseville, CA USA; 4 School of Medicine, University of California San Francisco, San Francisco, CA USA; 5 Department of Obstetrics and Gynecology, Brigham and Women’s Hospital, Massachusetts General Hospital, Boston, MA USA; 6 Department of Radiology and Biomedical Imaging, University of California San Francisco, San Francisco, CA USA

## Abstract

**Background:**

Invasive lobular carcinoma (ILC) is the second most common subtype of breast cancer, comprising 10–15% of cases. Due to its diffuse growth pattern, conventional imaging techniques have decreased sensitivity for ILC. While breast magnetic resonance imaging (MRI) is often recommended for ILC, its accuracy following neoadjuvant therapy is unknown. We evaluated the accuracy of post-treatment MRI and examined the impact on surgical outcomes.

**Patients and Methods:**

We retrospectively analyzed 129 patients with ILC who underwent neoadjuvant chemotherapy (NAC) or endocrine therapy (NET) and had post-treatment MRI. We considered a 0.5 cm difference between longest tumor diameter on MRI and pathologic tumor size to be discrepant. Tumor imaging phenotype was categorized as mass, non-mass enhancement (NME), or mass + NME. We evaluated concordance between imaging and pathology by tumor phenotype and associations with positive margin rates using Stata 18.0.

**Results:**

Post-treatment MRI underestimated tumor size in 52.5% of cases, was concordant in 25.3%, and overestimated in 22.2%. The presence of NME was associated with a higher rate of tumor size underestimation (62.5% versus 39.5% for mass only, *p* = 0.023); mean underestimation was 3.4 cm in those with NME. Underestimation was associated with higher positive margin rates following breast-conserving surgery (*p* = 0.018). Finally, among patients with complete imaging response on MRI, 93.3% had residual invasive disease on pathology.

**Conclusions:**

Following neoadjuvant therapy, post-treatment MRI frequently underestimates tumor size in ILC, particularly in tumors with NME. Surgeons should consider these imaging limitations when planning resection, which could improve surgical outcomes.

Accurate preoperative imaging is an important component of surgical planning for breast cancer to allow for appropriate treatment selection. Precise measurement of tumor size is crucial, as underestimation can lead to positive margins and need for re-excision, while overestimation can lead to unnecessarily excessive resection of breast tissue or mastectomy. However, conventional imaging techniques have limitations, and accuracy may vary across different types of breast cancer. This is particularly relevant for patients with invasive lobular carcinoma (ILC), where tumor characteristics make accurate imaging assessment more challenging. 

ILC is the second most common subtype of breast cancer, comprising 10–15% of all cases.^1^ Due to the absence of the cell adhesion protein E-cadherin, ILC grows in a more diffuse and infiltrating pattern compared with invasive ductal carcinoma (IDC).^2^ As a result, accurate assessment of disease extent can be challenging for ILC. Mammography is known to have limited sensitivity for ILC, often substantially underestimating the extent of disease.^3^ Obtaining clear margins after breast-conserving surgery (BCS) is a challenge in the management of ILC, and up to 60% of patients have positive margins after BCS, requiring either re-excision surgery or conversion to completion mastectomy.^4–7^

Because of these challenges, some guidelines recommend the use of breast magnetic resonance imaging (MRI) for the evaluation of ILC.^8–11^ While several studies show that breast MRI has higher accuracy than conventional imaging in ILC, the evidence that breast MRI is associated with improved surgical outcomes, such as lower positive margin rates and greater success of breast conservation, is less robust.^12–16^ Consequently, the utility of breast MRI in the preoperative evaluation of patients with early stage breast cancer remains a topic of debate.

One factor that might explain the inconsistent accuracy of breast MRI is variation in the imaging appearance of different breast tumors, termed tumor imaging phenotype. For example, tumors that appear to grow without the formation of a discrete mass may be described as “non-mass enhancement” on MRI, with this finding being associated with higher discordance between tumor diameter as measured by imaging compared with surgical pathology.^17^

Additionally, the increasing use of neoadjuvant therapy may impact the accuracy of preoperative imaging tools. Thus far, there is a paucity of data on the accuracy of breast MRI for patients with ILC who undergo neoadjuvant treatment. Addressing this knowledge gap is critical, as neoadjuvant therapies are increasingly used to downstage tumors, improve surgical outcomes, and allow for BCS in cases that might otherwise require mastectomy.^2,18^

To address this gap, we evaluated the accuracy of breast MRI in patients with ILC treated with either neoadjuvant chemotherapy (NAC) or neoadjuvant endocrine therapy (NET) by determining the size discrepancy between post-treatment MRI tumor size and pathologic tumor size. Our secondary objective was to investigate the impact of tumor imaging phenotype on the accuracy of MRI tumor size assessments. 

## Patients and Methods

### Study Population

**Fig. 1:**
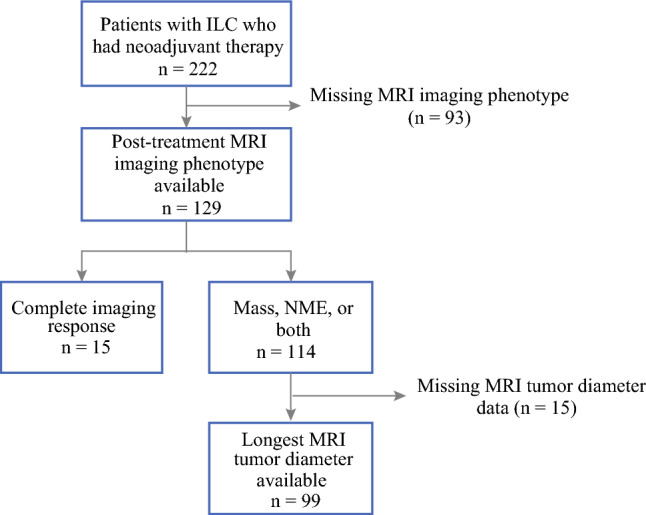
Patient cohort flow diagram

We queried a prospectively maintained institutional ILC database and identified 222 consecutive patients who received neoadjuvant therapy (NAC or NET). After excluding those with missing data, the study cohort consisted of 129 patients who underwent breast MRI after completion of neoadjuvant therapy with available imaging reports (Fig. 1).

### Variables

We collected data on patient demographics, tumor characteristics, hormone receptor status, imaging findings, and margin status. Tumor receptor subtype was defined by immunohistochemistry for estrogen receptor (ER), progesterone receptor (PR), and human epidermal growth factor-2 (HER2), along with fluorescence in situ hybridization for HER2, and based on post-treatment surgical specimens. ER and PR staining of ≥ 1% were considered positive; HER2 was considered amplified when 3+ by immunohistochemistry or overexpressed by fluorescence in situ hybridization. When available, highest Ki-67 was recorded on a continuous scale.

Clinical reports from pre-treatment and post-treatment breast MRI studies were reviewed for tumor imaging phenotype and total extent of disease. Tumor imaging phenotype was categorized as mass, non-mass enhancement (NME), or mass + NME on the basis of the American College of Radiology Breast Imaging Reporting and Data System (BI-RADS) nomenclature.^19^ Total extent of disease on MRI was the longest dimension of disease reported by the radiologist; in cases of multifocal tumors the total span of disease was measured. For cases that were reported to have both mass + NME, MRI images were retrospectively reviewed by a single breast radiologist to measure the total extent of disease including both mass and NME components. Pathologic staging was assigned on the basis of the American Joint Committee on Cancer 7th edition. Pathology reports were reviewed for invasive tumor size, margin status, and number of positive lymph nodes. Invasive tumor size was calculated according to standard clinical protocols by multiplying the number of involved specimen slices by average slice thickness. Positive margins were defined as ink on the tumor on the basis of guidelines published by the Society of Surgical Oncology (SSO) and American Society of Radiation Oncology (ASTRO).^20^ Lobular carcinoma in situ at a margin was not considered a positive margin; additionally, in this cohort no cases had concomitant ductal carcinoma in situ.

### Analysis Plan

We compared patient demographic and clinical characteristics between those receiving NAC and NET using chi-squared tests for categorical variables and *t*-tests for continuous variables. To assess the accuracy of MRI in estimating post-treatment tumor size, we compared the longest tumor diameter on post-treatment MRI with the tumor size reported on surgical pathology. We calculated size discrepancy between longest tumor diameter on MRI compared with longest tumor diameter on surgical pathology as pathology tumor size (cm) minus longest diameter on post-treatment MRI (cm). Size discrepancy was evaluated both as a continuous variable and categorically, where we considered a ± 0.5 cm discrepancy between MRI longest tumor diameter and pathology tumor diameter to be overestimation (pathology size–MRI longest diameter < − 0.5 cm) or underestimation (pathology size–MRI longest diameter > 0.5 cm).^21,22^ When post-treatment MRI longest tumor diameter was within 0.5 cm of pathology tumor size the MRI was considered to be concordant. We investigated factors associated with overestimation or underestimation of tumor size on imaging, including type of neoadjuvant therapy, tumor imaging phenotype, receptor subtype, number of positive nodes on pathology, and highest Ki-67. Additionally, we determined whether MRI accuracy was associated with surgical outcomes, including positive margin rates after BCS. In the BCS group, we compared mean size discrepancy in those with positive and negative margins.

The data were analyzed in Stata 18.0 using chi-squared tests to compare categorical variables,* t*-tests and analysis of variance (ANOVA) for continuous data, and multivariate logistic regression models. Two-tailed *p*-values < 0.05 were considered significant. Pearson correlation analysis was used to evaluate the relationship between post-treatment MRI longest tumor diameter and pathology tumor size.

## Results

### Patient Characteristics

Of 129 cases included, 84 patients (65.1%) received NAC and 45 patients (34.9%) received NET (Table 1). The mean age of the study cohort was 56.6 years (standard deviation [SD] 10.9). Compared with the NET cohort, patients who underwent NAC were younger (54.5 versus 60.6 years, *p* = 0.003), more often had triple negative (3.7% versus 2.3%) or HER2 positive tumors (13.4% versus 2.3%), and more often had grade 3 tumors (11.2% versus 0%). For the single patient in the NET group with triple negative ILC, pre-treatment diagnosis showed PR positivity on fine-needle aspiration; for the single patient with a HER2 positive tumor, pre-treatment receptor staining results were unavailable. Mean Ki-67 was significantly higher in the NAC group compared with the NET group (20.2% versus 10.2%, *p* = 0.002). Overall, there were more patients with stage 2 disease (46.5%) compared with stage 1 (22.0%) or stage 3 (29.1%), with no statistically significant difference between the treatment groups. However, mean tumor diameter at baseline was longer in the NAC group.

**Table 1:** Patient characteristics of the overall study cohort of patients with invasive lobular carcinoma (ILC)

Characteristic	Overall	Neoadjuvant chemotherapy (NAC)	Neoadjuvant endocrine therapy (NET)	*p*-Value
ILC patients [*n* (%)]	129	84 (65.1%)	45 (34.9%)	
Average age [years (SD)]^*n*^ = ^129^	56.6 (10.9)	54.5 (11.3)	60.6 (9.1)	0.003
Receptor subtype [*n* (%)]^*n*^ = ^125^				0.279
ER+PR+HER2−	88 (70.4%)	58 (70.7%)	30 (69.8%)	
ER+PR-HER2−	21 (16.8%)	10 (12.2%)	11 (25.6%)	
ER−PR−HER2−	4 (3.2%)	3 (3.7%)	1 (2.3%)	
HER2+	12 (9.6%)	11 (13.4%)	1 (2.3%)	
Grade [*n* (%)]^*n*^ = ^125^				0.021
1	37 (29.6%)	19 (23.8%)	18 (40.0%)	
2	79 (63.2%)	52 (65.0%)	27 (60.0%)	
3	9 (7.2%)	9 (11.2%)	0	
Number of positive nodes [*n* (SD)]^*n*^ = ^126^	2.4 (4.3)	2.1 (3.0)	2.8 (6.0)	0.348
Pathology tumor size [cm (SD)]^*n*^ = ^129^	4.6 (3.8)	4.7 (3.9)	4.3 (3.6)	0.610
Stage [*n* (%)]^*n*^ = ^127^				0.378
1	28 (22.0%)	15 (18.3%)	13 (28.9%)	
2	59 (46.5%)	41 (50.0%)	18 (40.0%)	
3	37 (29.1%)	23 (28.0%)	14 (31.1%)	
4	3 (2.4%)	3 (3.7%)	0 (0.0%)	
Highest Ki-67 [*n* (SD)]^*n*^ = ^66^	16.9 (15.3)	20.2 (15.8)	10.2 (11.8)	0.002
Longest tumor diameter on MRI				
Baseline [cm (SD)]^*n*^ = ^115^	4.9 (2.7)	5.4 (2.6)	3.8 (2.7)	0.003
Post-treatment [cm (SD)]^*n*^ = ^99^	3.5 (2.6)	3.8 (2.7)	3.0 (2.3)	0.143
Post-treatment imaging phenotype^*n*^ = ^114^				
Mass	45 (39.5%)	26 (36.6%)	19 (44.2%)	0.578
Mass + NME	25 (21.9%)	15 (21.1%)	10 (23.3%)	
NME only	44 (38.6%)	30 (42.3%)	14 (32.6%)	

### Tumor Imaging Phenotype on Breast MRI

Of the 129 patients, 114 (88.4%) had residual findings on post-treatment breast MRI while 15 patients (11.6%) had a complete imaging response. The mean post-treatment tumor diameter was 3.5 cm (SD 2.6 cm, range 0–12 cm). There was no significant difference in mean post-treatment tumor size on MRI between the NAC and NET groups (3.8 cm versus 3.0 cm, *p* = 0.143).

On post-treatment MRI, tumor imaging phenotype was mass only in 45 (39.5%) patients, mass + NME in 25 (21.9%), and NME only in 44 (38.6%) (Table 1). There was no significant difference in tumor imaging phenotype between the NAC and NET groups. Additionally, there was no difference in mean age, tumor size on pathology, number of positive nodes, tumor stage, tumor grade, receptor subtype, or highest Ki-67 by imaging phenotype (Table 2).

**Table 2:** Patient characteristics by tumor imaging phenotype

Characteristic	Mass	Mass + NME	NME	*p*-Value
Average age [years (SD)]^*n*^ = ^114^	57.4 (11.3)	57.5 (10.7)	54.9 (11.3)	0.484
Receptor subtype [*n* (%)]^*n*^ = ^110^				0.403
ER+PR+HER2−	29 (65.9%)	16 (69.6%)	32 (74.4%)	
ER+PR−HER2−	11 (25.0%)	2 (8.7%)	8 (18.6%)	
ER−PR−HER2−	1 (2.3%)	1 (4.3%)	0 (0%)	
HER2+	3 (6.8%)	4 (17.4%)	3 (7.0%)	
Grade [*n* (%)]^*n*^ = ^111^				0.358
1	8 (18.6%)	8 (33.3%)	15 (34.1%)	
2	31 (72.1%)	13 (54.2%)	27 (61.4%)	
3	4 (9.3%)	3 (12.5%)	2 (4.5%)	
Number of positive nodes [*n* (SD)]^*n*^ = ^111^	2.6 (5.5)	0.9 (1.6)	3.3 (4.5)	0.077
Pathology tumor size [cm (SD)]^*n*^ = ^114^	3.9 (3.3)	4.6 (4.1)	5.8 (4.2)	0.068
Overall stage [post-NAT, *n* (%)]^*n*^ = ^112^				0.084
1	11 (25.6%)	5 (20.0%)	5 (11.4%)	
2	18 (41.9%)	15 (60.0%)	22 (50.0%)	
3	13 (30.2%)	3 (12.0%)	17 (38.6%)	
4	1 (2.3%)	2 (8.0%)	0 (0%)	
Highest Ki-67 [*n* (SD)]^*n*^ = ^58^	17.0 (18.6)	22.4 (15.2)	13.7 (11.9)	0.158

### Accuracy of Breast MRI Overall

Among the cohort of 114 patients with residual findings on post-treatment MRI, 99 (86.8%) had available data on longest tumor diameter by MRI and pathology tumor size. Overall, there was a moderate correlation between post-treatment MRI longest tumor diameter and pathology tumor size (*r* = 0.5, *p* < 0.0001^23^). There was very strong correlation between MRI size and pathology tumor size for those with mass or mass + NME tumor phenotype (*r* = 0.8, *p* < 0.0001). However, there was no statistically significant correlation between MRI longest diameter and pathology tumor size in those with NME only (*r* = 0.3, *p* = 0.170).

Post-treatment MRI underestimated tumor size in over half the cohort, with size being underestimated in 52 (52.5%) patients, correctly estimated in 25 (25.3%) patients, and overestimated in 22 (22.2%) patients. Mean pathology tumor size was significantly larger in cases that were underestimated by post-treatment MRI (6.4 cm, SD 3.5) compared with cases that were correctly estimated (2.6 cm, SD 2.3) or overestimated (2.7 cm, SD 2.7; *p* < 0.001). Patients who received NET had a numerically higher proportion of tumor size underestimation than those who received NAC, but this did not reach statistical significance (59.5% versus 48.4% respectively, *p* = 0.107) (Table 3).

**Table 3:** Factors associated with tumor size discrepancy

Characteristic	Tumor size overestimated	Tumor size concordant	Tumor size underestimated	*p*-Value
ILC patients [*n* (%)]^*n*^ = ^99^	22 (22.2%)	25 (25.3%)	52 (52.5%)	
Average age [years (SD)]^*n*^ = ^99^	57.5 (12.3)	57.7 (10.7)	55.1 (10.9)	0.548
Type of therapy^*n*^ = ^99^				0.107
Neoadjuvant chemotherapy [*n* (%)]	18 (29.0%)	14 (22.6%)	30 (48.4%)	
Neoadjuvant endocrine therapy [*n* (%)]	4 (10.8%)	11 (29.7%)	22 (59.5%)	
Receptor subtype [*n* (%)]^*n*^ = ^95^				0.031
ER+PR+HER2−	14 (63.6%)	16 (69.6%)	38 (74.5%)	
ER+PR−HER2−	4 (18.2%)	1 (4.3%)	11 (21.6%)	
ER−PR−HER2−	0 (0%)	2 (8.7%)	0 (0%)	
HER2+	4 (18.2%)	4 (17.4%)	2 (3.9%)	
Grade [*n* (%)]^*n*^ = ^95^				0.689
1	6 (28.6%)	5 (20.0%)	15 (30.0%)	
2	12 (57.1%)	18 (72.0%)	32 (64.0%)	
3	3 (14.3%)	2 (8.0%)	3 (6.0%)	
Number of positive nodes [*n* (SD)]^*n*^ = ^97^	1.5 (2.6)	1.6 (2.6)	3.4 (6.0)	0.398
Pathology tumor size [cm (SD)]^*n*^ = ^99^	2.7 (2.7)	2.6 (2.3)	6.4 (3.5)	< 0.001
Stage [*n* (%)]^*n*^ = ^97^				0.003
1	9 (42.9%)	7 (28.0%)	2 (3.9%)	
2	6 (28.6%)	14 (56.0%)	28 (54.9%)	
3	5 (23.8%)	4 (16.0%)	19 (37.3%)	
4	1 (4.8%)	0 (0%)	2 (3.9%)	
Highest Ki-67 [*n* (SD)]^*n*^ = ^53^	15.1 (13.0)	21.1 (24.0)	18.0 (13.8)	0.733

Tumor size underestimation was associated with a higher proportion of stage 2 (54.9%) or stage 3 (37.3%) disease (*p* = 0.003). Rates of tumor size discrepancy varied by tumor receptor subtype, with higher rates of tumor size underestimation in hormone receptor positive HER2- cases compared with triple negative or HER2+ cases (*p* = 0.031). Of note, in the two triple negative ILC cases, post-treatment MRI tumor longest diameter was concordant with pathology tumor size. Among the ten cases of HER2+ ILC, post-treatment MRI overestimated tumor size in four (40.0%) cases. The rates of overestimation and underestimation did not differ significantly by age or tumor grade (Table 3).

### Accuracy of Breast MRI Stratified by Tumor Imaging Phenotype

**Fig. 2:**
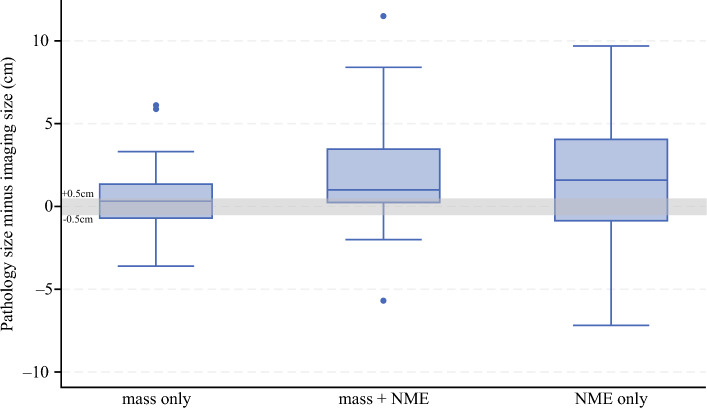
Box plot of the discrepancy between post-treatment MRI long diameter and pathology tumor size by imaging tumor imaging phenotype

**Fig. 3:**
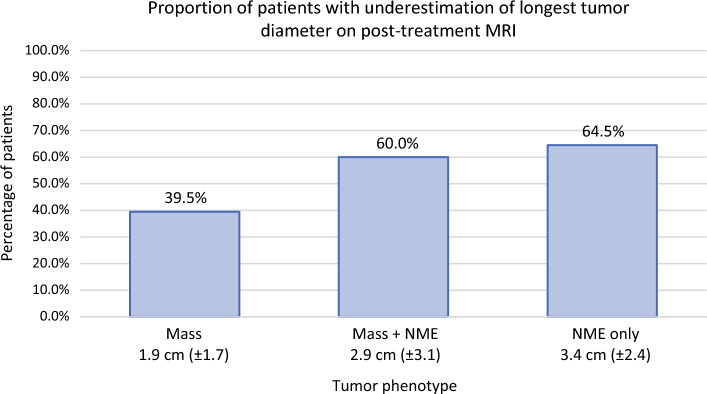
Percentage of patients for whom post-treatment MRI underestimated tumor size by tumor imaging phenotype (underestimation defined as MRI longest tumor diameter is ≥ 0.5 cm smaller than pathologic tumor size); values under each bar indicate the mean amount of tumor size underestimation (cm ± SD) for each tumor imaging phenotype category

Tumor imaging phenotype on post-treatment MRI was significantly associated with imaging accuracy, with NME being associated with a higher rate of tumor size underestimation (Fig. 2). MRI underestimated pathology size in 39.5% of cases with a mass, 60.0% of those with mass + NME, and 64.5% of those with NME only (Fig. 3). When comparing the proportion of tumor size underestimation across all patients, the presence of any NME (mass + NME or NME only) was associated with a significantly higher rate of underestimation compared with mass only cases (62.5% versus 39.5%, *p* = 0.023). After adjusting for tumor receptor subtype, patients with mass + NME had significantly higher odds of tumor size underestimation compared with those with mass only (OR 3.58, 95% CI 1.13–11.34, *p* = 0.030). Additionally, among the subset of patients with tumor size underestimation (*n* = 52), tumor imaging phenotype was more frequently mass + NME or NME only compared with mass only, although this was not statistically significant (67.3% versus 32.7%, *p* = 0.072).

When evaluating size discrepancy on a continuous scale, the degree of underestimation varied by imaging phenotype on MRI. Among the 63 patients where the MRI longest diameter was smaller than tumor size on pathology, post-treatment MRI underestimated true tumor size by an average of 2.7 cm (SD 2.5). This degree of underestimation was greatest in patients with a tumor imaging phenotype of NME only. Specifically, the mean size underestimation was 3.4 cm for NME only cases, compared with 1.9 cm for mass only and 2.9 cm for mass + NME cases (*p* = 0.125). When grouped by the presence of any NME (mass + NME or NME only), the degree of underestimation was greater compared with mass only cases (3.1 cm versus 1.9 cm), with the difference approaching statistical significance (*p* = 0.053).

When considering size discrepancy overall, including underestimation and overestimation, patients with any NME had significantly greater differences between MRI and pathology tumor size compared with those with mass only phenotype (1.7 cm versus 0.3 cm, *p* = 0.029). There was no significant difference in mean size discrepancy between the NAC and NET groups (1.0 cm versus 1.4 cm, *p* = 0.548). Among the 33 patients for whom post-treatment MRI longest tumor diameter was greater than pathology tumor size, the mean size discrepancy was 1.8 cm (SD 1.8). The mean size discrepancy in this group was not statistically different by tumor imaging phenotype, with mean overestimation of 1.4 cm for mass only, 1.7 cm for mass + NME, and 2.7 cm for NME only (*p* = 0.196).

### Impact of Breast MRI Accuracy on Surgical Outcomes

In this cohort, 67 (51.9%) patients underwent BCS as their first surgery. Among those, 51 patients had post-treatment MRI data available. MRI accuracy was significantly associated with risk of positive surgical margins. Patients with underestimation of disease on MRI had higher positive margin rates (72.0%) compared with those with concordant (26.7%) or overestimation of disease on MRI (45.5%, *p* = 0.018). Tumor size discrepancy was significantly greater in those with positive margins compared with those with negative margins (*p* = 0.007). Post-treatment MRI underestimated tumor size by an average of 1.3 cm (95% CI 0.4–2.2) in those with positive margins and overestimated tumor size by an average of 0.5 cm (95% CI −1.4 to 0.5) in those with negative margins.

Overall, 15 (11.6%) patients had a complete imaging response following neoadjuvant therapy (Table 4). Of these, 14 patients (93.3%) had residual invasive tumor on surgical pathology, with an average tumor size of 2.8 cm (range 0.1–9 cm). Only one patient (with HER2+ disease) had a complete pathologic response after NAC. In these 15 patients who had a complete imaging response, 8 underwent BCS, with 4 (50%) having positive margins.

**Table 4:** Characteristics of patients who had a complete imaging response following neoadjuvant therapy

Characteristic	Patients with complete imaging response (*n* = 15)
Average age [years (SD)]	57.9 (9.5)
Type of therapy	
Neoadjuvant chemotherapy [*n* (%)]	13 (86.7%)
Neoadjuvant endocrine therapy [*n* (%)]	2 (13.3%)
Receptor subtype [*n* (%)]	
ER+PR+HER2−	11 (73.3%)
ER+PR-HER2−	0 (0%)
ER−PR−HER2−	2 (13.3%)
HER2+	2 (13.3%)
Grade [*n* (%)]^*n*^ = ^14^	
1	6 (42.9%)
2	8 (57.1%)
3	0 (0%)
Number of positive nodes [*n* (SD)]	1.2 (1.7)
Pathology tumor size [cm (SD)]	2.8 (2.6)
Stage [*n* (%)]	
1	7 (46.7%)
2	4 (26.7%)
3	4 (26.7%)
4	0 (%)
Highest Ki-67 [*n* (SD)]^*n*^ = ^8^	15.9 (13.4)

## Discussion

In our study of patients with ILC treated with neoadjuvant therapy, we found that post-treatment MRI underestimated pathology tumor size in 52.5% of patients and overestimated size in 22.2% of patients. Among patients for whom tumor size was smaller on MRI than pathology, the average extent of underestimation was 2.7 cm. Underestimation was more frequent in cases with larger tumor size or NME. When considering overall size discrepancy, patients with NME had significantly greater MRI-pathology size mismatch compared with those with mass only imaging phenotype. Additionally, we found that tumor size underestimation on MRI was significantly associated with surgical outcomes, with higher rates of positive margins among those with tumor size underestimation. Finally, of the 15 patients with complete imaging response after neoadjuvant therapy, residual tumor remained in 14 (93.3%), suggesting that complete imaging response after NAT in ILC is an unreliable predictor of pathologic complete response.

Accurate assessment of disease extent for ILC remains challenging, and breast MRI is considered the most accurate imaging modality. Compared with mammography and ultrasonography, MRI assessment of tumor size demonstrates a stronger correlation with pathologic size, yet limitations remain.^21,22,24–32^ MRI underestimates ILC tumor size in 14–56% of cases, while overestimation occurs in 11–39%.^21,22,26,29^ However, direct comparison of studies can be challenging, as different size discrepancy thresholds have been used to define underestimation and overestimation.

Our study used a 0.5 cm size discrepancy as a threshold, similar to two prior studies on ILC. Hovis et al. studied 56 patients with ILC and found that MRI underestimated tumor size in 18% of patients and overestimated in 36%. On average, ILC span on MRI overestimated tumor size by 1.6 mm, with a range of −1.8 mm to 4.9 mm, whereas conventional imaging underestimated ILC span by 7.8 mm.^21^ Similarly, McGhan et al. found that tumor size was underestimated in 14% of patients while overestimation occurred in 31% of patients by MRI.^22^ Compared with these studies, our study demonstrated a much higher proportion of MRI tumor size underestimation (54.1%). Importantly, these prior studies did not evaluate patients who received NAT. Our findings suggest that preoperative systemic therapy has a major impact on the accuracy of breast MRI in those with ILC.

Indeed, the accuracy of MRI for estimating residual tumor size in ILC following NAT has not been well-studied. Post-treatment changes, including fibrosis and necrosis, can further complicate tumor size estimation and impact surgical planning.^33^ One study including 44 patients with ILC who received neoadjuvant therapy and obtained a preoperative MRI found that 70.5% of cases received the appropriate surgery, either BCS or total mastectomy, on the basis of MRI findings. However, among patients who underwent initial BCS, the rate of subsequent total mastectomy due to positive margins was much higher among patients who received neoadjuvant therapy compared with those who did not.^34^ In a study by Straver et al. that included 25 patients with ILC, MRI indicated the incorrect surgical treatment in 48% of patients with ILC who received neoadjuvant therapy, however, tumor imaging phenotype was not considered.^35^ To our knowledge, our study is the first to evaluate MRI accuracy in ILC following neoadjuvant therapy stratified by tumor imaging phenotype. We previously reported on the accuracy of preoperative MRI for evaluating axillary nodal status following neoadjuvant therapy in a similar cohort of 79 patients with ILC. The overall accuracy of MRI for predicting nodal status after neoadjuvant therapy was low, ranging from 45.5 to 66.7%.^36^ These findings collectively highlight the broader limitations of post-treatment MRI in ILC.

We found that the presence of NME on preoperative MRI was associated with a higher risk of tumor size underestimation and overall size discrepancy. NME, which refers to areas on breast MRI that show contrast enhancement without a mass, occurs in 20–40% of ILC tumors, which is slightly higher than observed in IDC.^32,37^ Our findings are consistent with other investigators who have shown that the presence of NME confers greater measurement variability compared with a mass-like imaging phenotype.^21^ We also showed in a previous study that tumors with well-defined imaging phenotypes on MRI, such as solid and well-contained versus diffuse and infiltrative, have higher concordance between post-treatment imaging findings and pathology size.^17^

Additionally, multiple studies, mostly evaluating patients with IDC, have demonstrated that the presence of NME on preoperative MRI is associated with close or positive margins and need for re-excision in patients who underwent BCS.^38–42^ Yoon et al. evaluated 101 patients with ILC and found that the presence of NME on preoperative MRI was associated with positive resection margins, in addition to multifocality, axillary lymph node metastasis, and pathologic tumor size.^43^ Interestingly, when NME occurs for other tumor types such as ductal carcinoma in situ, it has been shown to be associated with overestimation of tumor size on MRI.^44^ This highlights the importance of subtype-specific and treatment-specific interpretation of imaging studies.

Our findings suggest potential important clinical implications regarding the interpretation of post-treatment MRI in patients with ILC. Given the observed discrepancies between MRI and pathologic tumor size, especially in cases with NME, surgeons should consider incorporating imaging phenotype into preoperative planning for patients with ILC. In select cases, the surgical approach may be modified to resect a larger area of tissue to account for the anticipated size discrepancy if NME is observed on preoperative MRI following neoadjuvant therapy. Additionally, future work evaluating molecular differences between ILC of varying imaging phenotypes may provide insights into tumor biology.

This study had several limitations. Although we utilized a prospectively maintained institutional database, the retrospective nature of our study inherently carries the potential for selection bias. Additionally, our sample was relatively small, particularly for patients with NME, which could limit the generalizability of our findings. Finally, longest tumor diameter is unlikely to be the most accurate MRI measure of tumor size, although this is commonly used in clinical practice for surgical planning.

In summary, in patients with ILC who receive neoadjuvant therapy, the risk of tumor size underestimation on post-treatment MRI is substantial, particularly in the setting of NME on MRI. Patients with these features should be counseled on the increased risk of positive margins and possible need for re-excision. Surgeons should consider adopting a more aggressive resection strategy, including the use of oncoplastic approaches, during BCS to account for the anticipated size discrepancy.^45,46^ Additional studies are needed to further optimize breast MRI and imaging studies for ILC as well as to understand the drivers for these imaging features.
